# Concurrent hospitalization of a married couple presenting with distinct symptoms but diagnosed with a life-threatening infectious disease

**DOI:** 10.1590/0037-8682-0134-2022

**Published:** 2022-08-05

**Authors:** Eduardo Atsushi Osawa, Alexandre Toledo Maciel

**Affiliations:** 1 Imed Group, Departamento de Pesquisa, São Paulo, SP, Brasil.; 2 Hospital São Camilo Unidade Pompéia, Unidade de Terapia Intensiva, São Paulo, SP, Brasil.

**Keywords:** Botulism, Foodborne, Neuroparalytic syndrome

## Abstract

We described the cases of a married couple hospitalized for distinct symptoms and developed a neuroparalytic syndrome with rapid progression. In Case 1, a 75-year-old woman was admitted for abdominal pain, diarrhea, and blurred vision. The patient developed acute respiratory failure, ptosis, and ophthalmoplegia. She died on day 15 because of an acute abdomen. In Case 2, her husband, a 71-year-old man, was admitted for diplopia. The patient developed abdominal distension and slurred speech. Later, he developed bilateral ptosis, ophthalmoparesis, and mydriasis. Botulism was suspected, and both patients received botulinum antitoxin. Our male patient survived but underwent prolonged rehabilitation.

## INTRODUCTION

Botulism is a rare infectious disease that presents with the rapid progression of the neuroparalytic syndrome. Here, we described the cases of a married couple who had concurrent hospitalization for distinct symptoms and were eventually diagnosed with foodborne botulism. Despite being a well-known entity, one of our patients developed a complicated course, which raised the hypothesis of a different underlying mechanism. Therefore, we performed a literature review in PubMed to compare and contrast the clinical and laboratory features with our case reports.

## CASE REPORTS

### Case 1

A 75-year-old woman presented to the emergency department (ED) with a 1-day history of abdominal pain, diarrhea, vomiting, weakness, dysarthria, and blurred vision. The patient medical history was significant for arterial hypertension, diabetes mellitus, and an episode of intestinal subocclusion secondary to adhesions, for which surgical treatment was performed. The patient vital signs were as follows: blood pressure, 150/90 mmHg; heart rate, 144 beats/min; respiratory rate, 23 breaths/min; and temperature, 36.4 °C. Upon physical examination, the patient was in distress due to abdominal pain, bowel sounds were absent, and the abdomen was tender on palpation without guarding or rebound. Abdominal computed tomography (CT) revealed moderate distension of the gastric chamber, jejunum, and proximal ileum, containing areas of air-fluid levels. In addition, an abrupt change in caliber was identified in the left iliac region at the site of the previous enterectomy; however, no obstructive factors were identified. Laboratory tests demonstrated a normal leukocyte count (9,740/mm^3^), slightly impaired renal function (serum creatinine: 1.5 mg/dL (normal: 0.5 to 0.9 mg/dL), and a low C-reactive protein concentration of 2.6 mg/dL (normal: < 5 mg/L). The surgical team adopted a conservative approach comprising intravenous fluid administration, antibiotics (ceftriaxone and metronidazole), and stomach decompression. In the ED, the patient developed acute respiratory failure requiring endotracheal intubation and was transferred to the intensive care unit (ICU). 

The patient received low-dose vasopressor therapy and light sedation to enable endotracheal tube tolerance. She then developed bilateral ptosis, ophthalmoplegia, and apnea despite being able to move all limbs and obey commands. Lumbar cerebrospinal fluid (CSF) examination results were normal. At this stage, her husband was admitted to the ICU and developed similar neurological abnormalities. Their next of kin reported that both patients ingested expired meat pie one day before the admission. Therefore, foodborne botulism was suspected, and botulinum antitoxin was administered on day 2. Blood samples from both patients and leftovers were sent to a specialized Brazilian diagnostic center for the detection of neurotoxins. 

The patient had significant fluctuations in blood pressure and heart rate, requiring an intermittent infusion of vasopressors and antiarrhythmic agents. On day 4, she experienced an episode of severe bradycardia followed by pulseless electrical activity, for which one cycle of cardiopulmonary resuscitation was delivered. This event was caused by severe dysautonomia.

Dexmedetomidine was administered to inhibit autonomic dysfunction. The patient also had periods of constipation but was eventually able to move her bowels with laxatives and tolerate enteral feeding. Electroneuromyography revealed a presynaptic lesion at the neuromuscular junction. Weaning from mechanical ventilation was challenging; therefore, a tracheostomy was performed on day 9. Subsequently, she underwent a period of motor rehabilitation, was able to communicate by signal, sit out of bed, and had periods of supported standing.

**FIGURE 1:**
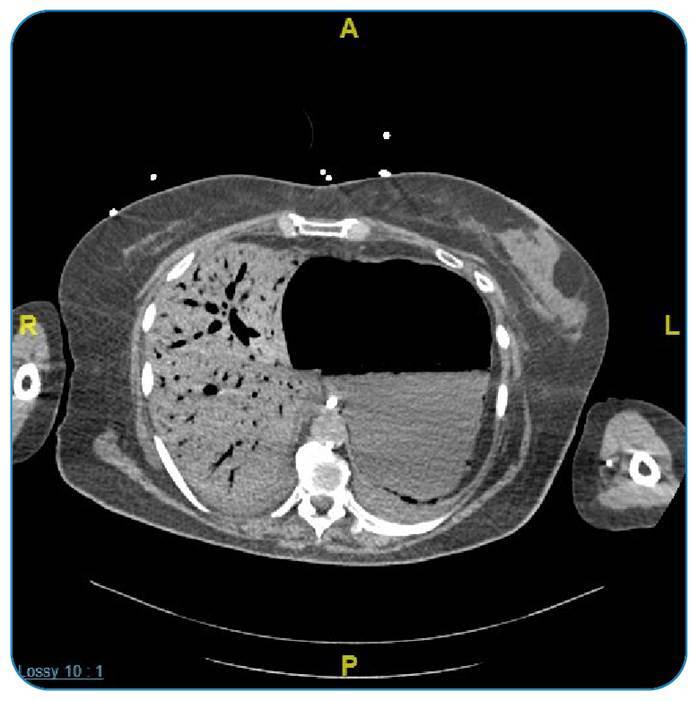
Abdominal computed tomography scan in Case 1 shows extensive portal venous gas, diffuse distension of small bowel loops, and long segments of intramural bowel gas.

On day 15, the patient developed sudden deterioration characterized by abdominal distension and circulatory shock. Abdominal computed tomography (CT) revealed extensive portal venous gas, diffuse distension of the small bowel loops, and long segments of intramural bowel gas (Figure 1). Mesenteric ischemia was suspected; however, the patient hemodynamic state rapidly deteriorated, preventing her from undergoing surgical treatment. Unfortunately, the patient died a few hours after the onset of the event.

### Case 2

A 71-year-old man presented to the ED with a complaint of diplopia which commenced 3 hours before admission. His pupils were equal and reactive to light, and his neurological examination was otherwise normal. A brain CT scan revealed no abnormalities, and he was admitted to the ICU with suspected acute ischemic stroke. During the following hours, the patient developed abdominal distension associated with nausea and vomiting. A nasogastric tube was inserted for stomach decompression; nevertheless, he complained of dry mouth and dysphagia. He then developed slurred speech along with tachypnea and desaturation secondary to the pooling of secretions in the upper airway, which required endotracheal intubation. His neurological examination findings remarkably changed as he developed ptosis, ophthalmoparesis, and dilated pupils, although the muscle strength of his upper and lower limbs was preserved. Lumbar CSF was normal. His wife's ongoing investigation prompted the administration of botulinum antitoxin on day 2. 

Over the following days, abdominal distension improved, and enteral nutrition was tolerated. Weaning from mechanical ventilation was difficult; therefore, a tracheostomy was performed on day 8. Electroneuromyography has also revealed presynaptic injury of the neuromuscular junction. In addition, levofloxacin was administered to treat tracheobronchitis. Laboratory investigations for botulinum neurotoxin in both serum and food samples returned positive results, thereby confirming the diagnosis of botulism. On day 32, the patient was transferred to a rehabilitation center using bi-level positive airway pressure ventilation via tracheostomy and receiving enteral nutrition via gastrostomy. 

## DISCUSSION

Botulism is a rare disease caused by a toxin produced by *Clostridium botulinum*, a Gram-positive, anaerobic, spore-forming bacterium. In contrast to the toxin, spores are resistant to high temperatures and germinate under specific set of conditions^1^. Four types of toxins are known to cause the disease in humans (A, B, E, and F), of which type A is responsible for greater disease severity and prolonged recovery time^2^. 

Our patient developed most of the distinctive features of botulism, a clinical syndrome characterized by symmetrical cranial nerve palsies, followed by descending muscle flaccid paralysis and respiratory arrest. The concurrent admission of both cases to the same unit enabled diagnosis and timely administration of the specific therapy. Moreover, the rapid repetitive nerve stimulation test performed during electroneuromyography showed a significant increase in the amplitude of the resting compound muscle action potential, indicating a presynaptic neuromuscular transmission injury. Such findings assist in the diagnosis and prevent patients from receiving empiric therapies for other conditions, such as stroke, Guillain-Barré syndrome, or myasthenia gravis.

Six clinical presentations have been documented: foodborne, wound, infant, adult intestinal toxemia, inhalational, and iatrogenic. Our female patient had previously undergone surgical treatment for intestinal subocclusion, a predisposing factor for adult intestinal toxemia. In this form, clostridia spores are ingested, colonize the lumen of the large intestine, and produce botulinum toxin *in situ* ^3^. As the clinical presentation is similar to other forms of botulism, it is challenging to distinguish adult intestinal toxemia from foodborne botulism. Previous investigations have reported findings indicative of adult intestinal toxemia, including prolonged elimination of spores or toxins and a high concentration of toxins in the feces concomitant with a low serum neurotoxin level^4^ ^,^ ^5^. In our setting, we could not send samples of stool or gastric contents for neurotoxin detection, thereby limiting our ability to further understand the etiology.

Laboratory investigations of botulinum neurotoxins are essential to confirm the diagnosis and suggest botulism’s etiology. However, the report can take several days to become available as specimens are injected intraperitoneally into mice, which are observed for signs of botulism^6^. In some cases, the identification of viable cells of *C. botulinum* in either food or stool may not be definitive in establishing the cause of the disease^7^. It is not uncommon for humans to ingest spores that do not undergo germination or toxin production. Thus, laboratory findings need to be interpreted in light of clinical and epidemiological information. 

In our patients, neurotoxin was detected in both serum and the expired food consumed, making foodborne botulism more likely. It is the most frequent form of botulism and results from the ingestion of preformed botulinum neurotoxins. The different complaints reported by our patients were initially believed to be coincidental occurrences of distinct medical conditions. Our male patient was admitted for diplopia, a rare initial manifestation^8^ ^,^ ^9^. In line with most reports, gastrointestinal symptoms preceded the onset of neurological symptoms in our female patient, and during the disease, constipation was a relevant issue experienced by both patients. Notably, our female patient had far greater disease severity, including severe autonomic dysfunction and cardiac arrest. Even after symptom subsidence and during rehabilitation, the patient developed an abdominal complication leading to death within a few hours. A possible explanation for this complicated course could be the persistence of intraluminal production of neurotoxin, in which case adult intestinal toxemia would be the underlying mechanism. If the diagnosis has been confirmed, further therapies could be considered, such as an additional dose of botulinum antitoxin and measures to eliminate intestinal colonization by *Clostridium* species^10^.

In conclusion, botulism is a life-threatening disease in which laboratory confirmation is not obtained immediately, and botulinum antitoxin must be delivered based on clinical grounds. Unfortunately, despite the provision of specific and supportive therapies, one of our patients developed a lethal complication, and the other survived with significant disabilities requiring prolonged rehabilitation.

### Ethical considerations

The patient’s next of kin provided consent for publication. The details of the case reports were anonymized.
